# An Exploratory Comparative Study of the Influence of Thai Massage on Postural Stability in Children with Overweight and Obesity

**DOI:** 10.3390/ijerph23010077

**Published:** 2026-01-06

**Authors:** Supapon Kaewsanmung, Vitsarut Buttagat, Ampha Pumpho, Phannarin Suwannarat, Petcharat Keawduangdee, Narongsak Khamnon, Niroat Chartpot

**Affiliations:** 1 Department of Physical Therapy, School of Integrative Medicine, Mae Fah Luang University, Chiang Rai 57100, Thailand; supapon.kae@mfu.ac.th (S.K.); ampha.pum@mfu.ac.th (A.P.); phannarin.suw@mfu.ac.th (P.S.); petcharat.kea@mfu.ac.th (P.K.); narongsak.kha@mfu.ac.th (N.K.); 2 Research Group on Smart Integrative Medicine and Technology Sustainability, Mae Fah Luang University, Chiang Rai 57100, Thailand; 3 Pa Tueng Sub-District Health Promoting Hospital, Mae Chan, Chiang Rai 57110, Thailand

**Keywords:** obesity children, postural control, postural balance, Thai massage

## Abstract

Background: Prolonged obesity in children can lead to a gradual decline in postural stability due to changes in biomechanics, musculoskeletal function, and neuromuscular control. Early interventions may help address these issues. This exploratory study examined the potential influence of Thai massage on postural stability in children with overweight and obesity. Methods: This study employed a quasi-experimental, comparative design and was conducted at the Pa Tueng Sub-district Health Promoting Hospital, Chiang Rai, Thailand. A total of 58 children meeting the criteria for overweight or obesity were systematically assigned to either the Thai massage group or the control group based on the order in which they were recruited. The Thai massage group received 45 min full-body Thai massage sessions combined with stretching exercises twice a week for six weeks (a total of 12 sessions), while the control group was advised to perform daily stretching exercises at home. Postural stability was assessed by measuring sway path length and sway velocity during double-leg and single-leg stances with eyes closed, both before and after the intervention. Results: While no statistically significant differences were found between the groups, the Thai massage group exhibited a non-significant trend toward reduced sway path length and sway velocity, whereas the control group showed a significant increase in both measures (*p* < 0.05), indicating a decline in postural stability over time. Conclusions: These findings suggest that Thai massage showed a potential trend toward stabilizing postural stability in children with overweight and obesity; however, the between-group differences were not statistically significant. As an exploratory study, further research with longer intervention durations and follow-up assessments is needed to determine whether clinically meaningful effects can be established.

## 1. Introduction

Postural equilibrium, often termed postural stability, is fundamental for maintaining proper body alignment and enabling smooth movement during both static and dynamic activities, such as sitting, standing, and walking. Maintaining an upright posture requires the coordinated integration of sensory inputs from the vestibular, somatosensory, and visual systems, along with effective motor control. Several factors influence postural stability in children, including age, sensory processing efficiency, and overall physical fitness levels [1].

There is substantial evidence linking excessive body mass to postural instability and altered weight distribution [2]. Studies utilizing multifractal analysis have indicated that overweight children experience greater plantar pressure, reduced mechanoreceptor sensitivity, lumbar hyperlordosis, and shortened gastrocnemius–soleus muscles. These biomechanical changes contribute to an anterior displacement of the center of mass, leading to difficulties in maintaining balance, especially in visually demanding tasks [1,2,3,4]. Additionally, research by D’Hondt et al. (2011) [5] and Maktouf et al. (2020) [6] suggest that prolonged childhood obesity can progressively deteriorate postural stability over time due to musculoskeletal adaptations, such as decreased joint flexibility and reduced muscle strength.

As postural instability worsens, children with obesity are at a heightened risk of falls, increasing their likelihood of fractures. Pediatric fractures are prevalent and can result in complications such as premature growth plate closure and skeletal deformities, potentially leading to long-term mobility issues [7,8]. Furthermore, impaired postural control can negatively affect motor coordination and movement efficiency, making children less inclined to participate in physical activities. This reluctance to engage in movement may further contribute to a sedentary lifestyle, perpetuating the cycle of obesity [9].

Thai massage is a traditional bodywork method that integrates deep manual pressure along designated meridian lines with stretching maneuvers [10,11]. This integration of stretching and deep manual pressure is specifically relevant to the biomechanical deficits identified in overweight children [12,13]. The stretching maneuvers may directly counteract the shortened gastrocnemius–soleus muscles and decreased joint flexibility, while the deep manual pressure may enhance mechanoreceptor activity and refine the crucial somatosensory input required for effective balance control [11]. It has been recognized for its ability to enhance body flexibility, walking efficiency, and balance in older adults [10] and improve foot sensitivity in individuals with diabetes [11]. Moreover, it has been applied as a therapeutic approach to address musculoskeletal conditions and enhance proprioception, which is crucial for postural control [11,14]. Given its potential effects on the somatosensory system, we hypothesize that Thai massage may help attenuate the decline of postural stability in children with overweight and obesity. Given the limited evidence in this population, the present exploratory comparative study was conducted to examine the potential influence of Thai massage on postural sway and postural control during functional stance tasks.

## 2. Materials and Methods

### 2.1. Study Design and Population

This study employed a quasi-experimental, exploratory comparative design. A total of 58 children, aged 8 to 13 years, classified as overweight (85th–< 95th percentile) or obese (≥95th percentile) according to age- and sex-specific BMI growth charts, were enrolled. Participants were recruited from schools in Chiang Rai, Thailand, using convenience sampling. They were subsequently assigned to either the Thai massage (TM) group or the control (CT) group using alternating allocation (i.e., sequential assignment to each group in the order of recruitment). Inclusion criteria required participants to have a body mass index (BMI) at or above the 85th percentile. Children were excluded if they exhibited conditions that could affect their participation, such as cardiac or pulmonary diseases, neurological disorders, visual or hearing impairments, recent fractures in the lower extremities, fever above 37.5 degrees Celsius, unstable blood pressure, skin or joint inflammation, or physical deformities. The study protocol was approved by the Human Research Ethics Committee of the Chiang Rai Provincial Public Health Office (CRPPHO 157/2565) on 29 December 2022. Written informed consent was obtained from all participants, and parental consent was secured for each child’s involvement in the research.

Sample size estimation was performed using an effect size derived from Hemmati et al. (2016) [15]. Their study reported center-of-pressure (COP) velocity values, a velocity-based postural sway parameter conceptually comparable to the sway velocity outcome used in the present study. The massage-plus-stretching group demonstrated a mean COP velocity of 0.169 mm/s (SD = 0.061), whereas the stretching-only group showed 0.124 mm/s (SD = 0.040), yielding a between-group mean difference of 0.045 and a pooled standard deviation of approximately 0.052 (Cohen’s *d* ≈ 0.87). Based on this effect size, an a priori power analysis using an independent-samples *t*-test in G*Power software (Version 3.1.9.6) (Heinrich Heine University Düsseldorf, Germany) (α = 0.05, power = 80%) indicated that 22 participants per group were required. Allowing for an anticipated 30% dropout, the target enrollment was increased to 29 participants per group.

**Figure 1:**
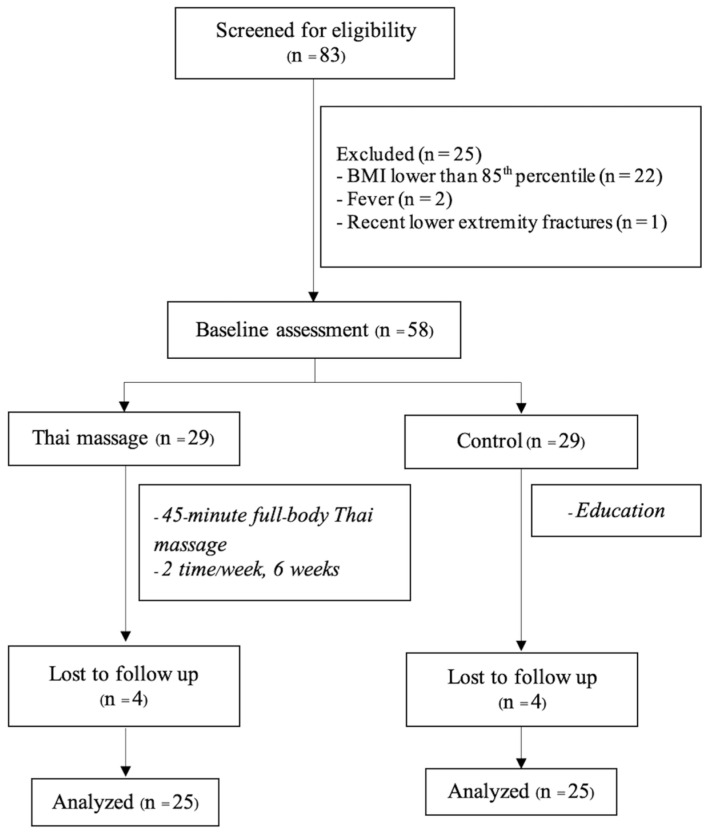
The CONSORT flow diagram.

The CONSORT flow diagram is presented in Figure 1. A total of fifty individuals completed the study, comprising 29 males and 21 females. Baseline characteristics of participants, including age, height, weight, BMI, and percentile distributions, are presented in Table 1. The two groups were comparable at baseline.

### 2.2. Setting

Pa Tueng Sub-district Health Promoting Hospital, Mae Chan, Chiang Rai, Thailand.

### 2.3. Research Protocols

**Figure 2:**
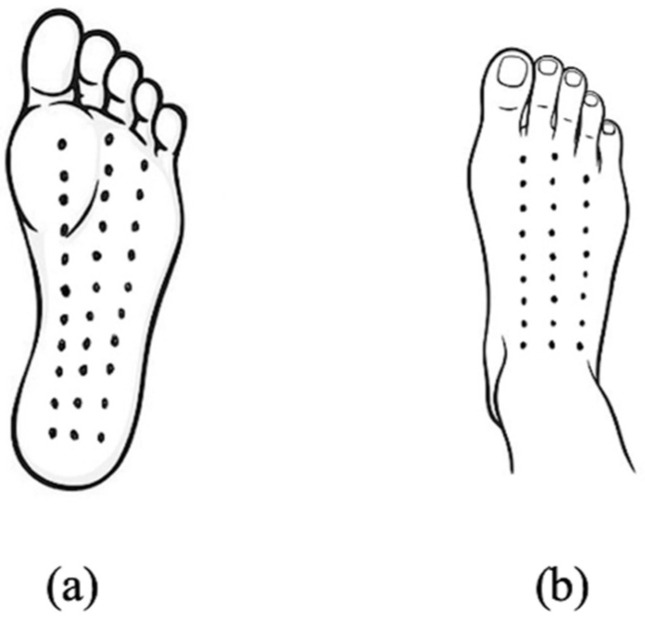
Feet massage line (**a**) Palmar, (**b**) Dorsal.

**Figure 3:**
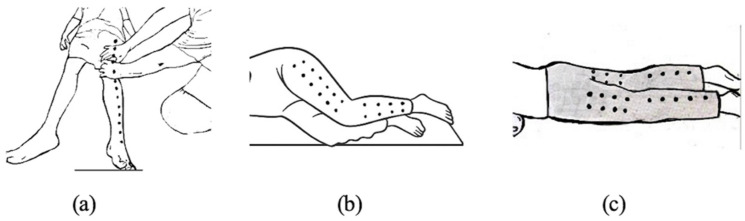
Lower limb massage line (**a**) Anterior (**b**) Lateral (**c**) Posterior.

**Figure 4:**
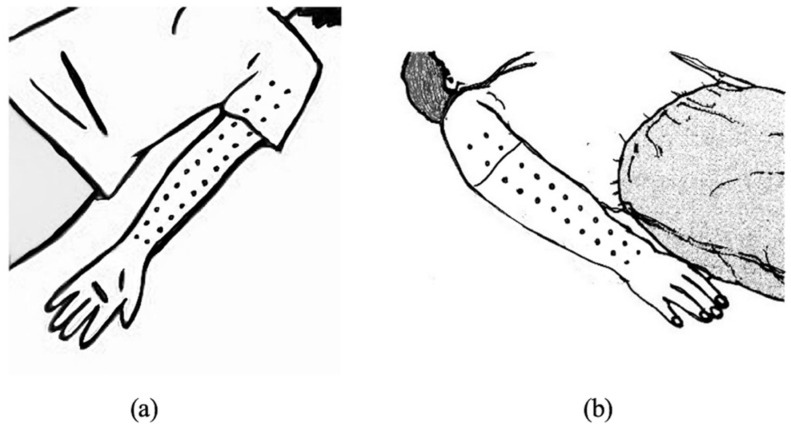
Upper limb massage line (**a**) Palmar (**b**) Dorsal.

**Figure 5:**
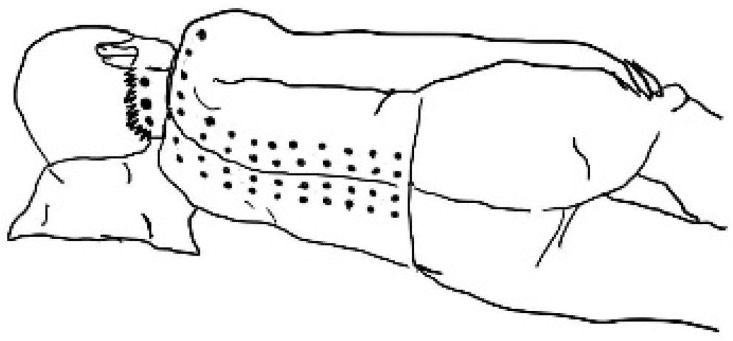
Back, Neck, Shoulder massage line.

**Figure 6:**
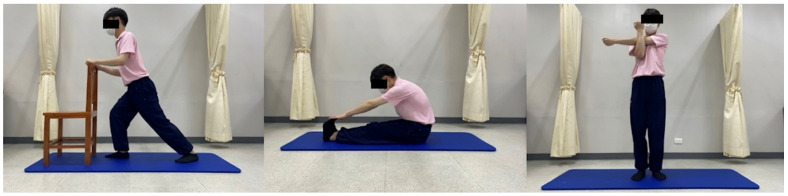
Muscle stretching for control group.

Children identified as overweight or obese who agreed to participate in the study were initially screened to ensure they met the established eligibility criteria. Those who qualified were then asked to provide demographic information and were divided into two groups. The experimental group (TM group) received a 45 min full-body Thai massage session. This intervention involved applying firm thumb pressure along the “meridian lines” (Figure 2, Figure 3, Figure 4 and Figure 5), covering regions such as the neck, back, upper and lower limbs, and feet. Each pressure point was held for 5 s, repeated three times, and complemented by gentle stretches targeting the calf, hamstring, quadriceps and scapular muscles to enhance relaxation and flexibility (Figure 6) [10]. In contrast, the control group performed a standardized set of stretching exercises targeting the calf, hamstring, quadriceps, and scapular muscles. Each stretch was held for 30 s and repeated three times per muscle group. Participants were instructed to perform the routine once daily for at least two days per week, independently and without supervision. This home-based stretching program was designed as a minimal-intervention comparator that did not involve therapist contact or manual stimulation. The stretching positions provided to participants are illustrated in Figure 6. The Thai massage sessions were administered by a certified massage therapist with over 5 years of professional experience. These sessions took place twice weekly for a total of 6 weeks, with each session lasting 45 min.

### 2.4. Outcome Measure

Postural balance was evaluated under two conditions: double-leg stance and single-leg stance, both performed with eyes closed. Sway path length and sway velocity were measured as key parameters. Participants stood barefoot on a force platform (Kistler Instrument, Winterthur, Switzerland), which captured data at a sampling rate of 100 Hz. For the double-leg stance, participants adopted an open stance with their feet slightly apart and turned outward, while keeping their hands resting alongside their torso. In the single-leg stance condition, participants balanced on their preferred leg, maintaining the position for the duration of the test. To minimize extraneous movement, participants were instructed to remain as still as possible with their eyes closed. Each participant performed three 30 s trials, with 10 s rest intervals between trials. The average of the three trials was calculated and used for analysis [15,16].

### 2.5. Statistical Analysis

Descriptive statistics were applied to summarize participants’ characteristics. The normality of the data was examined using the Kolmogorov–Smirnov test. Baseline characteristics such as age and weight were expressed as mean ± standard deviation (SD) and compared between groups using the independent sample *t*-test. Categorical variables were reported as frequencies and percentages, and comparisons between groups were performed using the chi-square test. Differences in outcomes between the two groups were analyzed using analysis of covariance (ANCOVA), with baseline values entered as covariates. Within-group changes from pre- to post-intervention were evaluated using the paired *t*-test. Effect Size Measures Partial eta-squared values (ηp^2^) were used as measures of effect size and magnitude for ANCOVA, interpreted as trivial (<0.01), small (0.01–0.06), moderate (>0.06–0.14), and large (>0.14) [17]. Cohen’s *d* was used as the measure of effect size for within-group (paired *t*-test) comparisons, interpreted as trivial (<0.2), small (0.2–0.5), moderate (>0.5–0.8), and large (>0.8) [18]. All analyses were performed using SPSS version 20 (IBM Corp., Armonk, NY, USA)., with the level of statistical significance set at *p* < 0.05.3.

## 3. Results

### Results of Outcome Measures

Regarding the between-group analysis, no statistically significant differences were observed between the Thai massage (TM) and control (CT) groups for any of the postural stability outcomes after adjusting for baseline levels (Table 2). However, exploratory within-group comparisons revealed distinct patterns over the 6-week period. In the CT group, both sway path length and velocity during double-leg stance with eyes closed significantly increased from baseline (*p* < 0.05), indicating a decline in stability. In the TM group, no such deterioration was observed; instead, there was a non-significant trend toward reduced sway measures. A similar pattern was observed during the single-leg stance with eyes closed (Table 3).

## 4. Discussion

This exploratory study is the first to examine the potential influence of Thai massage on sway path length and sway velocity in children with overweight and obesity. As reported in the results, no statistically significant between-group differences were observed for any sway parameters after adjusting for baseline levels. However, the divergent within-group trends provide noteworthy insights into the potential role of this intervention.

Specifically, children in the CT group experienced marked increases in sway velocity and sway path length over the 6-week period (within-group effect size Cohen’s *d* ≈ 0.4), reflecting a progressive decline in postural control. Conversely, children who received Thai massage showed a non-significant trend toward stabilizing these parameters (within-group effect size Cohen’s *d* ≈ 0.04). Although these changes did not reach statistical significance between groups, the direction of the effects suggests that Thai massage may have limited the deterioration that naturally occurred in the control group. Clinically, the stabilization of sway measures is relevant because increases in sway path length have been associated with higher fall risk and impaired sensorimotor integration in children with high BMI [3,19,20].

Within-group analyses further emphasize this pattern. The control group demonstrated statistically significant worsening in postural stability during eyes-closed double-leg stance (Cohen’s *d* ≈ 0.4), whereas the Thai massage group showed non-significant reductions in the same variables. A similar but less pronounced trajectory was observed in the single-leg stance condition, where the control group again showed a tendency toward increased sway, while the massage group showed small improvements (adjusted between-group effect size for single-leg stance: ηp^2^ ≈ 0.49–0.5).

Taken together, these divergent within-group trajectories help contextualize the non-significant adjusted between-group findings. Although statistical significance was not reached, the consistent direction of change, with worsening in the control group and stabilization or modest improvement in the massage group, suggests a potentially meaningful protective effect of Thai massage on balance performance in children with overweight and obesity.

To better understand these patterns, it is important to consider the physiological and neuromechanical pathways through which Thai massage may influence postural control. Thai massage involves the application of deep pressure along meridian lines, engaging multiple muscle groups throughout the body. Previous studies have demonstrated its ability to improve gait parameters, balance, and flexibility, particularly in older adults [9]. One of the key mechanisms underlying these benefits is its influence on the somatosensory system, which plays a critical role in proprioception and postural control [10,21]. Through targeted pressure and stretching, Thai massage may enhance proprioceptive feedback, thereby improving the body’s ability to sense joint position and movement, which is essential for maintaining balance. Additionally, massage therapy has been shown to modulate neuromuscular function by altering active muscle stiffness through changes in neurological activation [22]. This process may contribute to enhanced motor control and stability, particularly in individuals with compromised postural control due to excess body weight. Moreover, the application of deep pressure to muscles and joints increases blood circulation and stimulates mechanoreceptors, including muscle spindles and Golgi tendon organs, which are crucial for postural regulation [11]. These sensory and segmental adaptations facilitate better integration of postural feedback mechanisms, allowing for more effective adjustments to maintain balance. Given that individuals with overweight and obesity often exhibit impaired postural control due to excess body mass and altered neuromuscular function, the ability of Thai massage to enhance proprioceptive sensitivity, improve neuromuscular activation, and stimulate sensory receptors may help counteract these deficits [10,11].

As this study is the first to investigate the effects of Thai massage on sway path length and sway velocity in children with overweight and obesity, no directly comparable research is available to validate its effectiveness. However, the observed physiological influence aligns with mechanistic findings in related populations. For instance, Tatchananusorn et al. [14] reported that a single one-hour session of full-body Thai massage significantly enhanced gait parameters, including step length and stride length, as well as trunk and hamstring flexibility in healthy adults. These improvements in flexibility and gait mechanics align with our study’s mechanism to mitigate biomechanical deficits. In a similar vein, Ruan et al. (2024) [23] discovered that children with autism who underwent parent-administered Thai massage twice weekly over eight weeks (totaling 16 sessions) exhibited enhanced heart rate variability and stride length. This demonstrates the intervention’s capacity to induce positive physiological and movement-based changes in a pediatric population with compromised neuromuscular function. These findings collectively imply that Thai massage promotes physiological changes that enhance stability and overall movement control in diverse groups, lending indirect support to the protective effect observed in our overweight cohort.

This study has certain limitations that should be considered. First, the lack of randomization in group allocation may have introduced selection bias, potentially influencing the study’s validity. Future research should consider employing randomized methods to strengthen the reliability of the findings. Second, this study focused exclusively on children aged 8 to 13 years, which may limit the generalizability of the results to other age groups. Third, participants were not blinded to their group allocation, which may have introduced expectation or measurement bias. Future research should consider implementing blinding strategies to improve the reliability and objectivity of the findings. Fourth, the children’s daily physical load was not monitored or controlled during the intervention period. Variations in their regular physical activity may have influenced postural stability and partially contributed to the non-significant between-group results. Finally, the lack of standardization and monitoring of the stretching exercises performed by the control group is a limitation. Although participants received instructions and demonstration, the home-based stretching was performed according to individual preference and without adherence tracking. This may have resulted in variability in exercise intensity and frequency, potentially reducing the internal validity of between-group comparisons.

## 5. Conclusions

This exploratory study suggests that a six-week Thai massage program showed a potential trend toward stabilizing postural control in children with overweight and obesity. However, no statistically significant between-group differences were demonstrated. These findings provide a basis for future research with larger sample sizes and longer intervention durations to determine whether definitive, clinically meaningful effects can be established.

## Figures and Tables

**Table 1:** Baseline demographic of participants.

Characteristics	Control Group (*n* = 25)		Massage Group (*n* = 25)		*p* Value
	Mean ± SD (Min–Max)	Median (Q1, Q3)	Mean ± SD (Min–Max)	Median (Q1, Q3)	
Age (years)	10.76 ± 1.13 (9–13)	11 (10, 11.50)	10.28 ± 1.42 (8–13)	10 (9, 11)	0.19
Height (cm)	149.64 ± 10.23 (127–165)	151.00 (144.00, 157.50)	147.56 ± 10.88 (130–174)	146 (137, 156)	0.49
Weight (kg)	57.06 ± 13.27 (36–82)	55.6 (47.00, 69.50)	53.93 ± 13.86 (36.50–93)	50 (45.10, 59.75)	0.42
Body mass index (kg/m^2^)	25.22 ± 4.09 (20.09–32.37)	24.56 (21.41, 28.96)	24.46 ± 3.64 (19.63–32.31)	23.91 (21.64, 26.17)	0.49
Number of overweight, *n* (%) (85th–<95th percentile)	5 (20)		6 (24)		0.73
Number of obese, *n* (%) (≥95th percentile)	20 (80)		19 (76)		0.73
Number of males, *n* (%)	14 (56)		15 (60)		0.77

**Table 2:** Comparison of Post-Test Measures Between the TM and CT Groups After Adjusting for Baseline Outcome Values (ANCOVA).

Variable	Control	TTM	Mean Difference (95% CI)	*p* Value, ηp^2^
**Eyes closed with double stance**				
Sway path length (mm)	240.96 ± 56.14	237.91 ± 56.13	3.05 (−30.98–37.08)	0.86, 0.05
Sway velocity (mm/s)	12.02 ± 2.76	11.90 ± 2.77	0.12 (−1.56–1.79)	0.89, 0.04
**Eyes closed with single stance**				
Sway path length (mm)	1340.24± 275.94	1203.53 ± 274.04	136.71 (−174.16–447.58)	0.36, 0.50
Sway velocity (mm/s)	66.86 ± 13.86	60.15 ± 13.77	6.71 (−8.90–22.32)	0.37, 0.49

**Table 3:** Comparison of Outcome Measures Between Baseline (Pre-Test) and Post-Test (6 Weeks) Assessments in the TM and CT Groups (paired *t*-test).

Variables	Group	Pre-Test	Post-Test	*p* Value, Cohen’s *d*
**Eyes closed with double stance**				
Sway path length (mm)	Control	179.18 ± 50.08	209.35 ± 56.22	0.002, 0.40
	Massage	275.51 ± 113.84	269.52 ± 94.02	0.68, 0.04
Sway velocity (mm/s)	Control	8.95 ± 2.50	10.45 ± 2.81	0.002, 0.40
	Massage	13.66 ± 5.68	13.46 ± 4.70	0.78, 0.04
**Eyes closed with single stance**				
Sway path length (mm)	Control	1135.38 ± 350.31	1278.43 ± 300.17	0.21, 0.33
	Massage	1255.70 ± 306.67	1227.30 ± 362.29	0.74, 0.06
Sway velocity (mm/s)	Control	56.67 ± 17.48	63.79 ± 15.01	0.21, 0.33
	Massage	62.66 ± 15.36	61.33 ± 18.15	0.75, 0.06

## Data Availability

The data used to support the findings of this study contain sensitive information about participants and are therefore not available in a public repository. Researchers seeking access to the raw datasets may submit a formal request to the corresponding author, subject to ethical review and approval for legitimate research use.

## Abbreviations

The following abbreviations are used in this manuscript:

TM	Thai massage group
CT	Control group
ANCOVA	Analysis of covariance
